# Understanding evolution in Poales: Insights from Eriocaulaceae plastome

**DOI:** 10.1371/journal.pone.0221423

**Published:** 2019-08-20

**Authors:** Ashwini M. Darshetkar, Mandar N. Datar, Shubhada Tamhankar, Pan Li, Ritesh Kumar Choudhary

**Affiliations:** 1 Agharkar Research Institute, Pune, Maharashtra, India; 2 Savitribai Phule Pune University, Pune, Maharashtra, India; 3 College of Life Sciences, Zhejiang University, Hangzhou, China; International Centre for Genetic Engineering and Biotechnology, INDIA

## Abstract

In this study, we report the plastome of *Eriocaulon decemflorum* (Eriocaulaceae) and make an effort to understand the genome evolution, structural rearrangements and gene content of the order Poales by comparing it with other available plastomes. The size of complete *E*. *decemflorum* plastome is 151,671 bp with an LSC (81,477bp), SSC (17,180bp) and a pair of IRs (26,507 bp). The plastome exhibits GC content of 35.8% and 134 protein-coding genes with 19 genes duplicated in the IR region. The Eriocaulaceae plastome is characterized by the presence of *accD*, *ycf1* and *ycf2* genes and presence of introns in *clpP* and *rpoC1* genes which have been lost in the Graminid plastomes. Phylogenomic analysis based on 81 protein-coding genes placed Eriocaulaceae sister to Mayacaceae. The present study enhances our understanding of the evolution of Poales by analyzing the plastome data from the order.

## Introduction

The order Poales contains 15 families [1] and over 20,000 species, representing about one-third of monocots [2]. The order also includes many economically important crops, such as rice (*Oryza sativa* L.), wheat (*Triticum aestivum* L.), maize (*Zea mays* L.), millets, bamboo and lots of ecologically important species that dominate modern Savanna and Steppe vegetation [2]. Poales can be simplified into five major groups viz. Bromeliads, Cyperids, Xyrids, Restiids, and Graminids [2,3,4]. The order has been studied for genome evolution, and ancient polyploidy events wherein transcriptome data was generated for representatives of each clade of the order [5,6]. As far as the phylogenomic studies are concerned, most of the studies are available for the Graminids, focusing on Poaceae because of its ecological, evolutionary, and economic importance [7,8]. Also, amongst Poales, the highest number (i.e., 396) of plastid genomes have been generated for the family Poaceae (https://www.ncbi.nlm.nih.gov/genome/). The plastomes of Poaceae have undergone several evolutionary events, such as inversions (28 kb inversion between *trnG-UCC* and *rps14* region, <1kb in the *trnT* sequence and a 6 kb in the *trnG-UCC*), complete loss of genes (*accD*, *ycf1* and *ycf2*) and intron losses in the genes *clpP*, *rpoC1* [7,8,9,10,11]. However, the genome information for other families from Graminids is sparse. Other than Poaceae, plastome sequence is available only for Joinvilleaceae [12]. Also, very few plastomes are available from Bromeliads (2 published, one unpublished) and Cyperids (3, Cyperaceae) (S1 Fig). No chloroplast genome has been sequenced for any member of Restiids until now. Besides, these major groups, no attempt has been made to understand the gene content, structural rearrangements, and genome evolution of order Poales as a whole.

The family Eriocaulaceae belongs to Xyrids of Poales and is sister to family Xyridaceae [2,3,4]. The family consists of ten genera and ca. 1400 species which are distributed throughout the tropics [13,14,15] and the family can be easily distinguished by characteristic capitulum or head inflorescence [16]. Ruhland [17] classified the family into two subfamilies Eriocauloideae and Paepalanthoideae comprising of two and eight genera, respectively. The members of Eriocaulaceae inhabit a variety of habitats like marshy or aquatic to terrestrial and xeric habitats. Moreover, they also comprise of both annuals and perennials [18]. *Eriocaulon* L. (subfamily Eriocauloideae) is the largest genus of Eriocaulaceae and exhibits cosmopolitan distribution [13,18,19]. Taxonomy of this genus has remained a challenge for taxonomists due to high intraspecific variations and limited interspecific differences [20,21,22]. Several studies have been conducted to understand relationships between the family Eriocaulaceae, including both morphological and molecular techniques [13–15,18–19,23–25]. However, all these studies include a wider sampling of the subfamily Paepalanthoideae but very few from Eriocauloideae. Molecular studies mainly included nuclear and chloroplast markers such as ITS, *trnL*-*F*, and *psbA*-*trnH* intergenic spacer [13–15,19,25,26]. Diaz Pena [26], for the first time, included plastome sequences to understand phylogeny and biogeography of the genus *Paepalanthus* subg. *Platycaulon*. However, the study did not mention accession numbers for the plastomes. Also, one *Eriocaulon* plastome (*E*. *sexangulare* L., MK193813), first for the genus, has been reported recently [27] but not yet available in public database. In spite of the availability of these plastome sequences, no attempts were made to understand the gene content, structural rearrangements, and genome evolution in the family concerning the evolution of order Poales.

In China, the genus *Eriocaulon* is represented by 35 species, 13 of which are endemic [28]. Some of the species have considerable use in Traditional Chinese Medicine [29–31]. *Eriocaulon decemflorum* Maxim. an important medicinal plant is distributed from China to Japan, Korea, and the Far East of Russia [28,32–34]. The species occurs in rice fields, marshy places, and mountain slopes at an altitude of 1600–1700 m [28]. The species has also been tested for its antibacterial activity against *Staphylococcus aureus* and *Pseudomonas aeruginosa* [35]. As per the recent assessment, the species has been listed as vulnerable in China [36]. In the present study, the assembly, annotation, and analyses of complete plastome of *E*. *decemflorum* Maxim. is reported. Attempts were also made, for the first time, to understand the position, structural arrangements, and evolution within Poales with the insights received from the *Eriocaulon* plastome.

## Materials and methods

### Sampling, DNA extraction, and sequencing

Fresh leaf samples of *Eriocaulon decemflorum* were collected from Mt. Dayang, Jinyun County, Zhejiang Province, China (August 2017, Voucher No. *X*.*L*. *Xie 170189*). Voucher specimens were deposited at the herbarium of Zhejiang University (HZU). The total DNA was extracted using Plant DNAzol Reagent (LifeFeng, Shanghai) according to the manufacturer's protocol from approximately 20 mg of the silica-dried leaf tissue. The high molecular weight DNA was sheared (yielding ≤ 800 bp fragments) and the quality of fragmentation was checked on an Agilent Bioanalyzer 2100 (Agilent Technologies). The short-insert (500 bp) paired-end libraries preparation and sequencing were performed by the Beijing Genomics Institute (Shenzhen, China). The sample was pooled with others and run in a single lane of an Illumina HiSeq X10 with a read length of 150 bp.

### Assembly, annotation and comparative analyses

The quality of reads was checked using software FastQC v. 0.11.7. [37]. Adapters and ends were trimmed with Cutadapt 1.16 [38], a Linux based software and Trimmomatic v 0.38 was used to filter the raw reads and to get high-quality clean reads [39]. De-novo genome assembly was carried out with curated reads using the software NOVOPlasty v.2.7.1 [40]. Forward and reverse reads with a read length of 150 bp and an average insert size of 300 bp were used for assembly. The default k-mer value of 39 was given in the configuration file. The seed input was an *rbcL* (ribulose-1,5-bisphosphate carboxylase/oxygenase) sequence of *Eriocaulon compressum* Lam. (EU832954). Since no reference plastome exists for any species of Eriocaulaceae, contigs could not be scaffolded by an automated method. Four contigs were produced after assembly. The contigs were then extended by mapping reads and other assembled contigs in Geneious Prime 2019.1.1 (www.geneious.com) until perfect overlap of at least 20 base pairs (bp) with other contigs or reads was obtained. This was repeated until the quadripartite plastome structure was completed. The orientation of IRs, LSC, and SSC regions was further confirmed by NCBI blast and graphic view. Genome annotation was performed with DOGMA [41] and using GeSeq–Annotation of Organellar Genome [42], an online tool of CHLOROBOX (https://chlorobox.mpimp-golm.mpg.de/geseq.html). For tRNAs prediction, additional tools such as ARAGORN v1.2.38 and tRNAscan-SE v2.0 were used. Sequences of *Typha latifolia* L. and *Ananas comosus* (L.) Merr. from Bromeliads were used as the references for annotation. The circular map of plastid genome was constructed by using OGDRAW [43]. The annotation was confirmed again with Geneious prime 2019.1.1 (www.geneious.com).

Reputer [44] was used to identify and locate forward, reverse, compliment, and palindromic sequences in the plastome of *Eriocaulon decemflorum* with n ≥ 30 and sequence identity ≥ 90. Microsatellite markers were identified using MISA [45] with minimal iterations of ten, five, four, three, three and three for mono-, di-, tri-, tetra-, penta- and hexa-nucleotide respectively. Microsatellite composition and positions in *E*. *decemflorum* were also compared with those of *Typha latifolia* (Typhaceae, Bromeliad), *Ananas comosus* (Bromeliaceae, Bromeliad), *Joinvillea ascendens* Gaudich. ex Brongn. & Gris (Joinvilleaceae, Graminid), *Anomochloa marantoidea* Brongn. (Poaceae, Graminid), *Carex neurocarpa* Maxim., *Carex siderosticta* Hance, *Hypolytrum nemorum* (Vahl) Spreng. (Cyperaceae, Cyperid) and *Musa textilis* Née (Musaceae, Zingiberales). Sizes of complete plastomes, inverted repeats, locations of IR/SSC junctions and arrangement of genes adjacent to IR/SSC borders were also analyzed for these genomes. Aforementioned genomes were also compared for gene content using MultiPipMaker [46] with annotation of *E*. *decemflorum* as a reference. Gene orders were examined by pair-wise comparison between *Eriocaulon*-*Typha* (a member of Bromeliad clade), *Eriocaulon*-*Hypolytrum* (a member of Cyperid clade) and *Eriocaulon*-*Anomochloa* (a member of Graminid clade).

### Phylogenomic analyses

The phylogenetic tree was constructed using 81 Coding DNA sequences (CDS) of the plastid genome. Most of the analyses were performed using the CIPRES Science Gateway [47]. The sequences were aligned using MAFFT v7.402 [48]. Maximum Likelihood (ML) analyses were performed using IQ-TREE v. 1.6.7 [49] using GTR+F+R4 model. Ingroup consisted of 57 taxa in total belonging to Bromeliaceae (1), Typhaceae (1), Eriocaulaceae (1), Cyperaceae (3), Joinvilleaceae (1) and Poaceae (50, representing all subfamilies). Data for 19 taxa available from the study of Givnish et al. [5] was also included to have a representation of all families of the order. The outgroup was composed of ten taxa belonging to Zingiberales (S1 Table). The output tree was visualized in FigTree v. 1.4.2 (http://tree.bio.ed.ac.uk/software/figtree/).

## Results and discussion

### Genome assembly

The illumina sequencing generated 99,54,908 paired end reads. Both untrimmed and trimmed reads generated a similar number of contigs after assembly. The average organelle coverage was 20X. The largest contig scored 91.93% of total organelle genome.

### Genome organization and features

**Fig 1:**
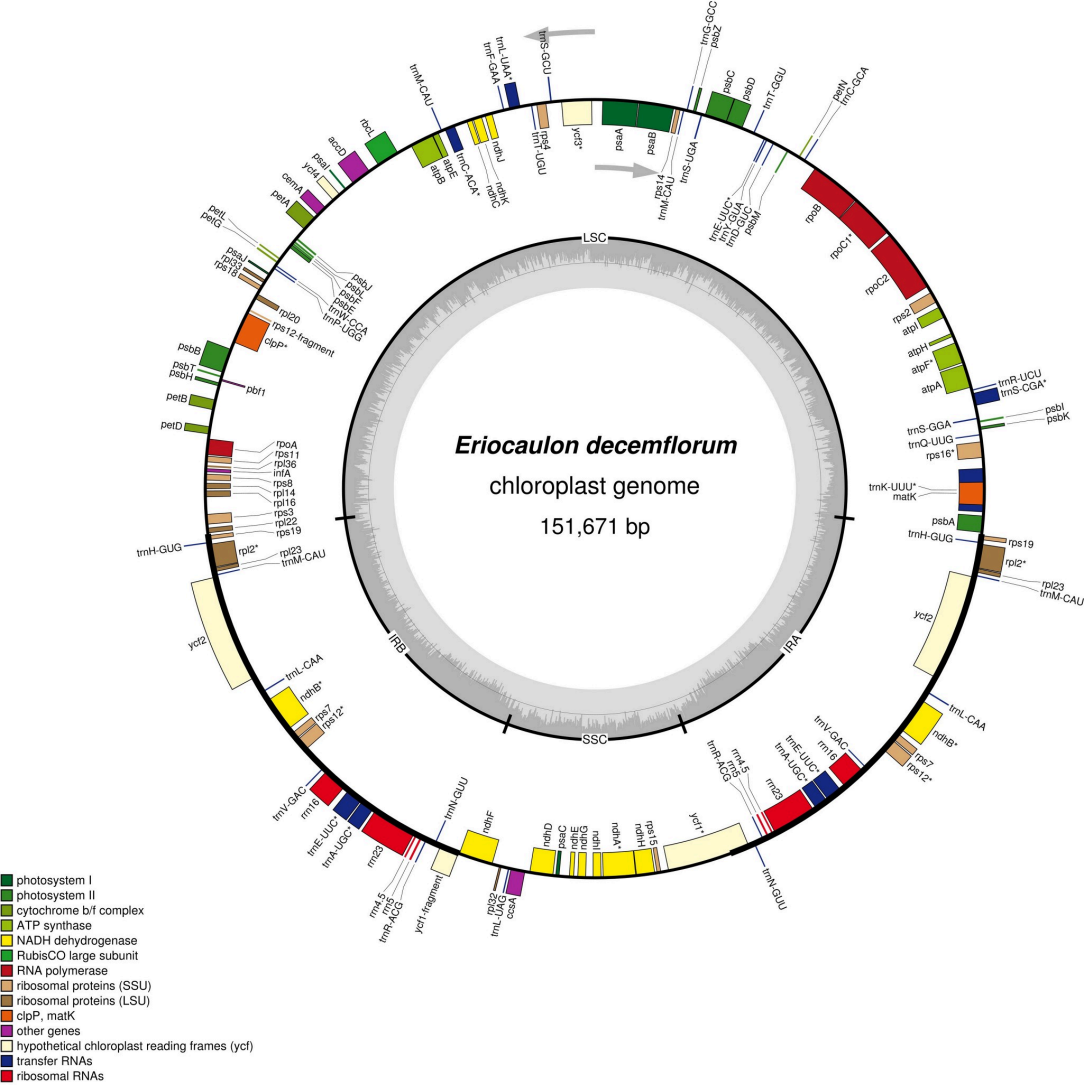
Plastome map of *Eriocaulon decemflorum*. Genes drawn inside the circle are transcribed clockwise, and those outside are counter-clockwise. Genes belonging to different functional groups are shown in different colors. The innermost circle denotes GC content across the plastome. The asterisks indicate genes which contain intron(s).

The plastome of *Eriocaulon decemflorum* exhibits a typical quadripartite structure, with an LSC (81,477bp), SSC (17,180bp) and a pair of IRs (26,507bp) (Fig 1, Table 1). The size of the complete plastome is 151,671bp (Fig 1, Table 1). The GC content of the whole plastome is 35.8%. GC contents of LSC, SSC and IR regions are 32.6%, 27.8%, and 43.2%, respectively. IR region exhibited more GC content. Higher GC content in the IR region is due to high GC content in the rRNA genes. IR region exhibits four rRNA genes containing 52.9% of GC content.

**Table 1:** Comparison of major features of *Eriocaulon decemflorum* and eight other plastid genomes.

Species→	*Eriocaulon decemflorum*	*Typha latifolia*	*Ananas comosus*	*Joinvillea ascendens*	*Anomochloa marantoidea*	*Musa textilis*	*Carex neurocarpa*	*Carex siderosticta*	*Hypolytrum nemorum*
Characters↓									
Genbank accession no.	MK639364	NC013823	NC026220	NC031427	GQ329703	NC022926	NC036037	NC027250	NC036036
Size (bp)	151671	161572	159636	149327	138412	161347	181397	195251	180648
LSC length (bp)	81477	89140	87482	85526	82274	88016	103711	102460	95644
SSC length (bp)	17180	19652	18622	12907	12162	18989	8476	8981 8981	8150
IR length (bp)	26507	26390	26766	25447	21988	27171	34605	41905	38427
Total no. of genes	134	131	141	122	145	133	129	127	137
No. of genes duplicated in IR	19	18	24	21	20	20	22	21	23
No. of genes with introns	16	18	18	17	17	20	17	20	18
% GC content	35.8	33.8	37.4	38.1	38.7	35.9	33.9	34.1	34.9

In the genome of *E*. *decemflorum*, a total of 134 genes was predicted, including 83 protein-coding genes, 31 tRNA genes, 4 rRNA genes duplicated in the IR region. List of genes is presented in Table 2. 19 genes are duplicated in IR and 16 genes contain introns, which include 10 protein-coding genes and 6 tRNAs.

**Table 2:** List of genes in the chloroplast genome of *Eriocaulon decemflorum*.

Category	Group of genes	Name of genes
Photosynthesis	Photosystem I	*psaA*, *psaB*, *psaC*, *psaI*, *psaJ*
	Photosystem II	*psbA*, *psbB*, *psbC*, *psbD*, *psbE*, *psbF*, *psbG*, *psbH*, *psbI*, *psbJ*, *psbK*, *psbL*, *psbM*, *psbN*, *psbT*, *psbZ*
	Cytochrome b/f complex	*petA*, *petB*, *petD*, *petG*, *petL*, *petN*
	ATP synthase	*atpA*, *atpB*, *atpE*, atpF*, *atpH*, *atpI*
	NADH-dehydrogenase	ndhA*, ndhB*(×2), *ndhC*, *ndhD*, *ndhE*, *ndhF*, *ndhG*, *ndhH*, *ndhI*, *ndhJ*, *ndhK*
	Large subunit of Rubisco	*rbcL*
Protein synthesis and DNA replication genes	Ribosomal RNAs	*rrn16 (×2)*, *rrn23 (×2)*, *rrn4*.*5(×2)*, *rrn5 (×2)*
	Transfer RNAs	trnA-UGC*, trnC-ACA*, *trnC-GCA*, *trnD-GUC*, trnE-UUC*, *trnF-GAA*, *trnfM-CAU*, *trnG-UCC*, *trnH-GUG (×2)*, *trnI-CAU*, *trnI-GAU*, trnK-UUU**, *trnL-CAA (×2)*, trnL-UAA*, *trnL-UAG*, *trnM-CAU (×2)*, *trnN-GUU (×2)*, *trnP-GGG*, *trnP-UGG*, *trnQ-UUG*, *trnR-ACG (×2)*, *trnR-UCU*, *trnS-GCU*, trnS-GGA*, *trnS-UGA*, *trnT-GGU*, *trnT-UGU*, *trnV-GAC (×2)*, *trnV-UAC (×2)*, *trnW-CCA*, *trnY-GUA*
	Small ribosomal unit	*rps11*, rps12* *(×2)*, *rps14*, *rps15*, rps16*, *rps18*, *rps19 (×2)*, *rps2*, *rps3*, *rps4*, *rps7 (×2)*, *rps8*
	Large ribosomal unit	*rpl14*, *rpl16*, rpl2* *(×2)*, *rpl20*, *rpl22*, *rpl23 (×2)*, *rpl32*, *rpl33*, *rpl36*
	RNA polymerase sub-units	*rpoA*, *rpoB*, rpoC1*, *rpoC2*
Miscellaneous group	Maturase	*matK*
	Protease	clpP**
	Acetyl-CoA-carboxylase sub-unit	*accD*
	Envelope membrane protein	*cemA*
	Component of TIC complex	*ycf1*
	c-type cytochrome synthesis	*ccsA*
Unknown	Hypothetical genes	ycf1*** *(×2)*, *ycf2 (×2)*, ycf3**, *ycf4*

* Genes containing one intron

** Genes containing two introns

*** Genes containing three introns

### Repeat and SSR analyses

**Fig 2:**
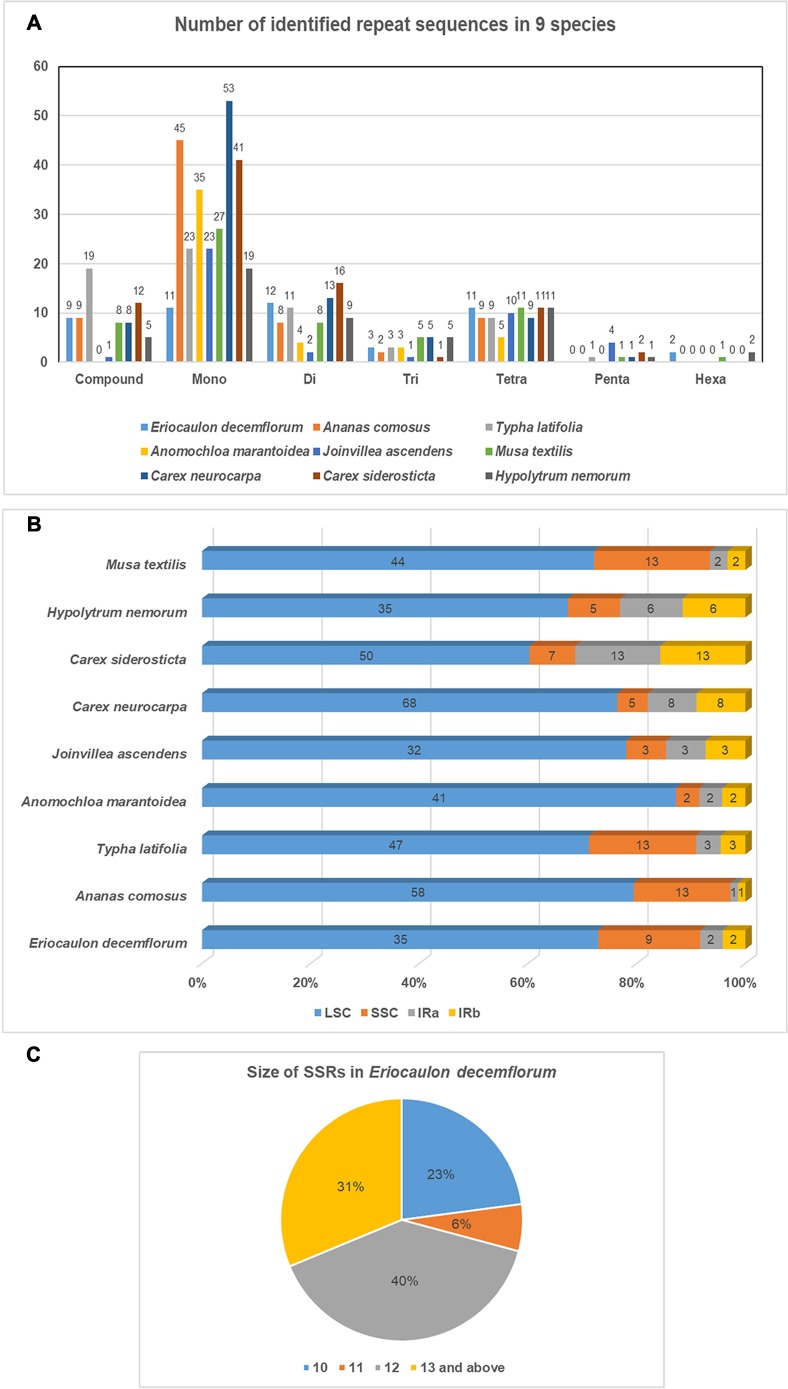
Number, position, and size of SSRs in *E*. *decemflorum*. A. Comparison of SSRs across nine genomes, B. Position of SSRs in nine compared genomes, C. Size of SSRs in *E*. *decemflorum*.

Plastome of *E*. *decemflorum* contains 21 forward, 17 palindromic, one complement and one reverse repeat (Table 3). Size of the repeats ranged from 30 to 150. Simple sequence repeats (SSRs) are another important type of repeats in the plastome used as a genetic marker because of their length polymorphism [50]. In total 48 SSRs were found in the genome of *E*. *decemflorum* including 11 mono, 12 di, three tri, 11 tetra, two hexa, and nine compound repeats. Comparison of several repeats identified in seven other genomes of Poales and one outgroup is presented in Fig 2. In LSC, SSC and IR regions 35, 9 and 2 SSRs were found respectively. All the SSRS found in *E*. *decemflorum* were AT-rich. The highest number of SSRs were found in *Carex neurocarpa* (Cyperaceae).

**Table 3:** Repeat sequences and their distribution in the *E*. *decemflorum* genome.

No.	Size	Type	Repeat 1 Start	Repeat 2 Start	Position
1	150	F	125014	125164	IR
2	86	F	88973	88997	IR
3	86	F	144065	144089	IR
4	65	F	88994	89018	IR
5	58	F	49336	49391	LSC
6	59	F	88976	89024	IR
7	59	F	144065	144113	IR
8	41	F	30211	30231	LSC
9	41	F	88994	89042	IR
10	41	F	42046	96451	LSC, IR
11	38	F	36287	38523	LSC
12	35	F	88976	89048	IR
13	35	F	144065	144137	IR
14	36	F	42051	117656	LSC, SSC
15	36	F	96456	117656	IR, SSC
16	31	F	7165	33980	LSC
17	31	F	8759	34973	LSC
18	30	F	30211	30251	LSC
19	30	F	37149	39373	LSC
20	30	F	42053	95653	LSC, IR
21	30	F	144077	144149	IR
22	150	P	107834	125014	SSC
23	86	P	88973	144065	IR
24	86	P	88997	144089	IR
25	65	P	88994	144065	IR
26	65	P	89018	144089	IR
27	59	P	88976	144065	IR
28	59	P	89024	144113	IR
29	41	P	88994	144065	IR
30	41	P	89042	144113	IR
31	41	P	42046	136656	LSC, IR
32	31	P	7165	43467	LSC
33	35	P	88976	144065	IR
34	35	P	89048	144137	IR
35	36	P	117656	136656	SSC, IR
36	32	P	33979	43467	LSC
37	32	P	73032	117656	LSC, SSC
38	30	P	42053	137465	LSC, IR
39	32	C	27952	114492	LSC, IR
40	30	R	27297	40657	LSC

### Comparative plastomic analyses

**Fig 3:**
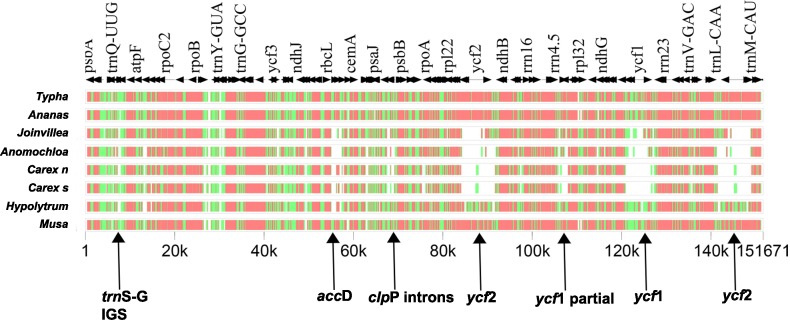
MultiPip analysis showing overall sequence similarity of plastid genomes based on complete genome alignment. Levels of sequence similarity are indicated by red (75±100%), green (50±75%), and white (<50%). The comparison included nine genomes using *Eriocaulon decemflorum* as a reference. Arrows indicate gene and intron losses. *Carex n* denotes *Carex neurocarpa*; *Carex s* denotes *Carex siderosticta*. Loss of *rpoC1* intron is not shown as it is only absent in *Anomochloa* among all compared genomes.

Among the nine compared genomes, *Anomochloa marantoidea* (Poaceae) has the smallest plastome (138,412 bp) while *Carex siderosticta* has the largest plastome (195,251 bp). When all eight genomes were compared with *Eriocaulon decemflorum* annotation as a reference. gene order and content were found to be conserved (Fig 3).

*Eriocaulon decemflorum* plastome exhibited two copies of the *ycf1* gene (one partial and one full length), which have been lost in the Graminids. The full-length *ycf1* gene has three introns in *E*. *decemflorum*. However, a functional *ycf2* gene is present in *E*. *decemflorum*, which also has been lost in the Graminids. In Bromeliads too, *ycf1* and *ycf2* genes are partially degraded [8]. Several indels have been reported in *ycf1*/*2* regions between *Ananas* and *Musa* [51].

#### Evolution of *accD* gene

The *accD* gene encodes one of the four subunits of acetyl co-A carboxylase enzyme required for the formation of malonyl-CoA from acetyl CoA, in the first step of fatty acid synthesis [52,53]. Its absence or partial degradation in some monocots (mostly in order Poales and family Acoraceae) is known [54]. Even though the gene is lost from plastome, a multifunctional nuclear-encoded enzyme is present in some monocot species [55,56]. Moreover, this region between *rbcL* and *psaI* is considered as a hotspot as it exhibits higher rates of mutations [57,58]. Katiyama and Ogihara [54] predicted the loss of the *accD* gene before the divergence of Poales and Commelinales. However, Konishi et al. [59] noted the presence of *accD* in Cyperids and Xyrids and hence proposed that the loss occurred later after Cyperid and Xyrid divergence. Harris et al. [60] however predicted loss of *accD* after the splitting of Eriocaulaceae and Xyridaceae. *Eriocaulon decemflorum* plastome exhibited the functional copy of the *accD* gene. In Bromeliads, partial degradation of *accD* was reported [8]. However, in *Musa*, *accD* is much longer as compared to Bromeliads [51]. Several studies have confirmed the presence of the *accD* gene in Cyperaceae [57,59,60]; however, sequences deposited on NCBI database lack *accD* gene, probably unannotated. No information is available for Restiid clade. Our results corroborate with those of Harris et al. [60], which supported the theory of gene loss after the Eriocaulaceae and Xyridaceae splitting.

#### Loss of introns

*rpoC1* encodes for the β′ subunit of RNA polymerase and consists of a single intron in most of the land plants. However, loss of *rpoC1* introns has also been reported in several lineages [61]. Katayama and Ogihara [54] noticed a loss of *rpoC1* introns in all the members of Poaceae and Restiid clade. However, Morris and Duvall [11] reported the presence of *rpoC1* intron in *Anomochloa* (Anomochloideae), one of the basal member of Poaceae. *rpoC1* intron has also been reported in the Bromeliads [7,8], and our study confirms the same in *Eriocaulon*, which is a member of Xyrids. However, further studies are required to trace the point of *rpoC1* intron loss in Poales.

The other protein-coding gene *clpP* of *Eriocaulon decemflorum* has maintained its two introns. The introns have also been reported for Bromeliad members [7,8,51] while they have been lost in the Graminids. Annotations provided for three Cyperaceae members on NCBI dataset, do not exhibit introns for both the genes. However, when we annotated these genomes keeping one of the three as a reference, both the genes exhibited the presence of introns.

**Fig 4:**
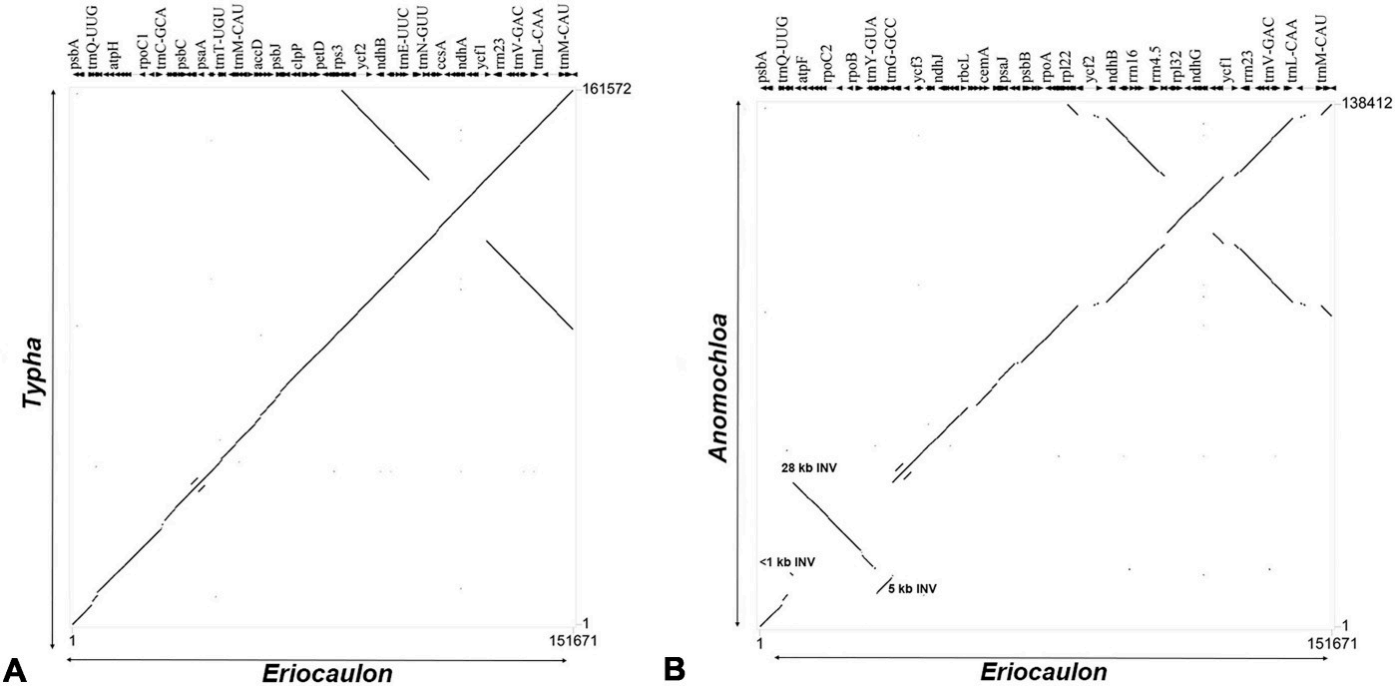
Percent identity plots. (A). *Eriocaulon decemflorum* compared to *Typha latifolia*. Numbers along the X-axis indicate the coordinates for *Eriocaulon* and along the Y-axis for *Typha*. (B). *Eriocaulon decemflorum* compared to *Anomochloa marantoidea*. Numbers along the X-axis indicate the coordinates for *Eriocaulon* and along the Y-axis for *Anomochloa*.

The gene order between Xyrids and Bromeliads appears to be conserved (Fig 4A) and between Xyrids and Graminids is characterized by three major inversions namely, 28-kb inversion between the *trnG-UCC*–*rps14* region, a 6-kb in the *trnG-UCC*–*psbD* region and the third in *trnT* and flanking region (Fig 4B). Two inversions were observed between *Eriocaulon* and *Hypolytrum* in LSC (44000–55000 bp region) and SSC (around 135000 bp region). The variations observed between Xyrids and Cyperids could be due to large genome size and longer inverted repeats reported from Cyperid genomes (S2 Fig).

### Contraction and expansion of IRs

**Fig 5:**
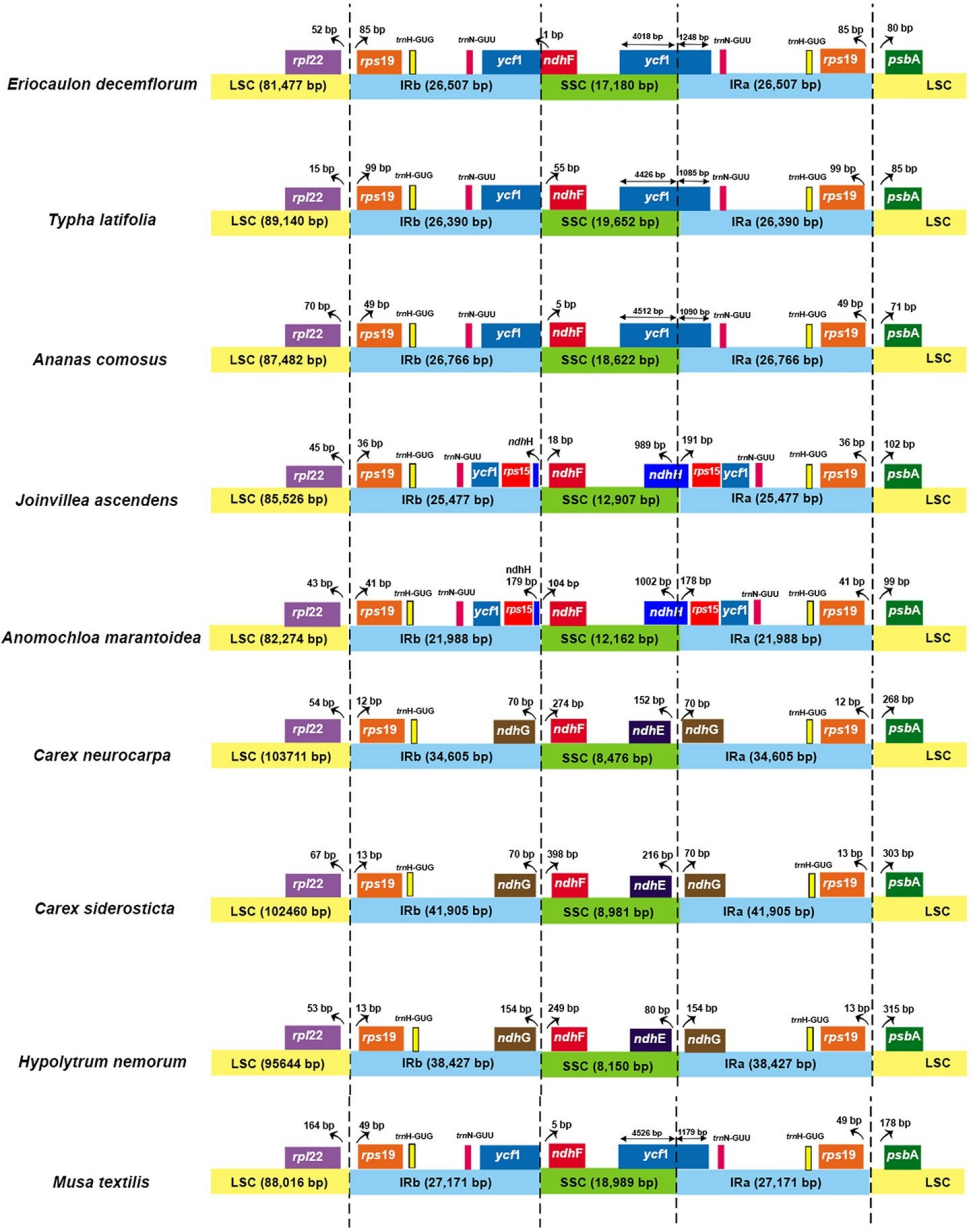
Comparison of plastome borders of LSC, SSC and IR regions. The extent of the inverted repeat (IR) in nine plastid genomes. Gene and IR lengths are not to scale.

The IRs in the plastomes are divided by four junctions viz. IRb/LSC, IRb/SSC, IRa/LSC, and IRa/SSC. The contraction and expansion of IR regions differ in various plant species. Such variation has already been observed in members of Poales [8,51]. All nine genomes were compared for their IR boundaries (Fig 5). All the compared genomes have expanded IRb/LSC and IRa/LSC to add both *trnH-GUG* and *rps19* to the IR region. The extent of IR expansion into the intergenic spacers between *rps19* and *rpl22* varies from 15 to 164 bp while between *rps19* and *psbA* varies from 71 to 315 bp. Three Cyperaceae members have long IRs, i.e. 34,605, 38,427 and 41,905 bp. IR/SSC junctions exhibit a lot of variations among members of Poales. Bromeliads, Xyrids, *Musa*, *Anomochloa* and *Joinvillea* have pseudogenized *ycf1* in the IR region at the IRb/SSC junction. In *Anomochloa* and *Joinvillea* (Graminids), IRb/SSC junction exhibits *rps15* and *ndhH* genes in the IR, which is characteristic to all grasses [7]. The Cyperaceae members exhibit *ndhG* gene at the IRb/SSC boundary. Poales have *ndhF* gene in SSC region at IRb/SSC junction ranging from 5 to 398 bp away from the junction. Only in *Eriocaulon*, it has 1 bp in the IR region. At the IRa/SSC junction, bromeliads, xyrids, and *Musa* have *ycf1* gene while the graminids have the *ndhH* gene. The Cyperids have *ndhE* and *ndhG* genes at this junction.

### Phylogenomic analyses

**Fig 6:**
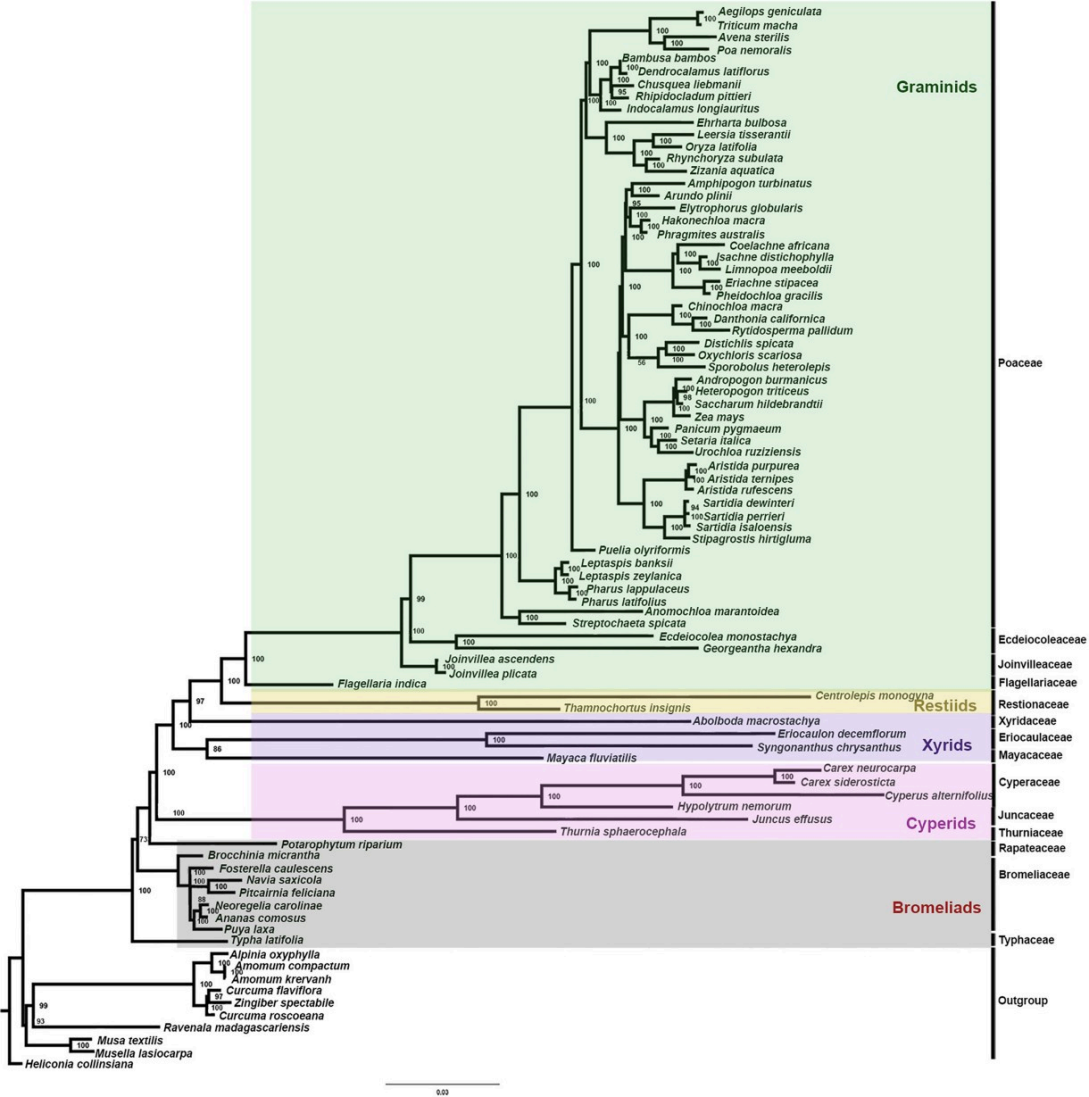
Maximum likelihood (ML) tree of protein-coding genes of Poales. Bootstrap values are indicated at the nodes.

The data matrix used for phylogenetic reconstruction was composed of 87 taxa, 77 belonging to Poales (representing 14 families) and 10 from Zingiberales as outgroup. ML analysis using IQTREE resulted in a tree having lnL of -613657.791. *Eriocaulon* and *Syngonanthus* appeared to be sisters with bootstrap value = 100 (Fig 6). Eriocaulaceae appeared sister to Mayacaceae with bootstrap value 86. However, Xyridaceae (*Abolboda*) appeared sister to the Restiid-Graminid clade which was in accordance with Han et al. [27]. Givnish et al. [5] tried to trace evolutionary history of the order based on plastome protein coding genes using both maximum parsimony (MP) and ML methods. MP analysis yielded Xyrids (Eriocaulaceae, Xyridaceae and Mayacaceae) as monophyletic with moderate bootstrap support. However, ML analysis resulted in Xyridaceae as sister to Restiid-Graminid clade and Mayacaceae and Eriocaulaceae appeared as sisters with strong bootstrap support. Recently, Mckain et al. [6] attempted to study evolutionary history as well as ancient polyploidy of Poales. They found that Eriocaulaceae (*Lachnocaulon*) and Xyridaceae (*Xyris*) were sisters but with very low support, and Mayacaeae (*Mayaca*) was not included in the analysis. Results obtained in our study are in accordance with the study of Givnish et al. [5] and Han et al. [27] where Eriocaulaceae appeared sister to Mayacaceae. Earlier studies have reported Xyrid clade as the most ambiguous clade in terms of its phylogenetic relationships [2–6]. In some studies, Xyridaceae and Eriocaulaceae were reported as sister families [3,4] while some suggested sister relationship of Eriocaulaceae and Mayacaceae [5,27]. However, the inclusion of more plastomes from all the three families will help in resolving relationships within this clade.

## Conclusion

In the last few years, plastomes have been widely used to study phylogeny and evolution in different plant groups, as well as for reconstructing the ancestral states of angiosperms. Important advances have also been made in our understanding of the relationship within the monocots [62]. Studies based on plastome data have shown that orchids and grasses together form a monophyletic group nested within the remaining angiosperms [63]. The present study enhances our understanding of the evolution of Poales by analyzing the plastome data from the order. Understanding relationships within Eriocaulaceae has always been difficult due to minute floral characters [18]. Hybridization events have also been reported for the family [36,64]. No attempts have been made to resolve species relationships and to understand evolutionary events, though *Eriocaulon* is the only wide-spread genus of the family. Deletion of genes like *accD*, *ycf1*, *ycf2* and intron losses in *clpP* and *rpoC1* genes are characteristic to graminids and were not found in other groups of Poales, i.e., Bromeliads and Cyperids. Our study shows that *Eriocaulon* plastome exhibits the presence of *accD*, *ycf1*, and *ycf2* genes, and also *clpP* and *rpoC1* introns similar to Bromeliads. *ycf1* is highly variable in terms of phylogenetic information at the level of species and has been shown to be subject to positive selection in many plant lineages [65]. In the present phylogenomic analysis, Eriocaulaceae is sister to Mayacaceae, which is in accordance with the previous study of Givnish et al. [5] and Han et al. [27]. However, the inclusion of more plastomes from Xyrids will further resolve the relationships between Xyridaceae, Mayacaceae, and Eriocaulaceae and will also help to understand evolution within Poales.

## Supporting information

S1 Fig Phylogenetic tree showing the major clades and families of Poales, after APG IV. Family names in green indicate the availability of plastome genomes. Numbers indicate available plastome genomes. Asterisks indicate the presence of available but unpublished genome.
(TIF)

S2 Fig Percent identity plot. *Eriocaulon decemflorum* compared to *Hypolytrum nemorum*. Numbers along the X-axis indicate the coordinates for *Eriocaulon* and along the Y-axis for *Hypolytrum*.
(TIF)

S1 Table NCBI accession numbers of the genomes included in phylogenomic analysis. (XLSX)
