# Supplementary figures and images for: Understanding evolution in Poales: Insights from Eriocaulaceae plastome
Source: PLoS One. 2019 Aug 20;14(8):e0221423. doi: 10.1371/journal.pone.0221423 (PMC6701780; doi:10.1371/journal.pone.0221423)

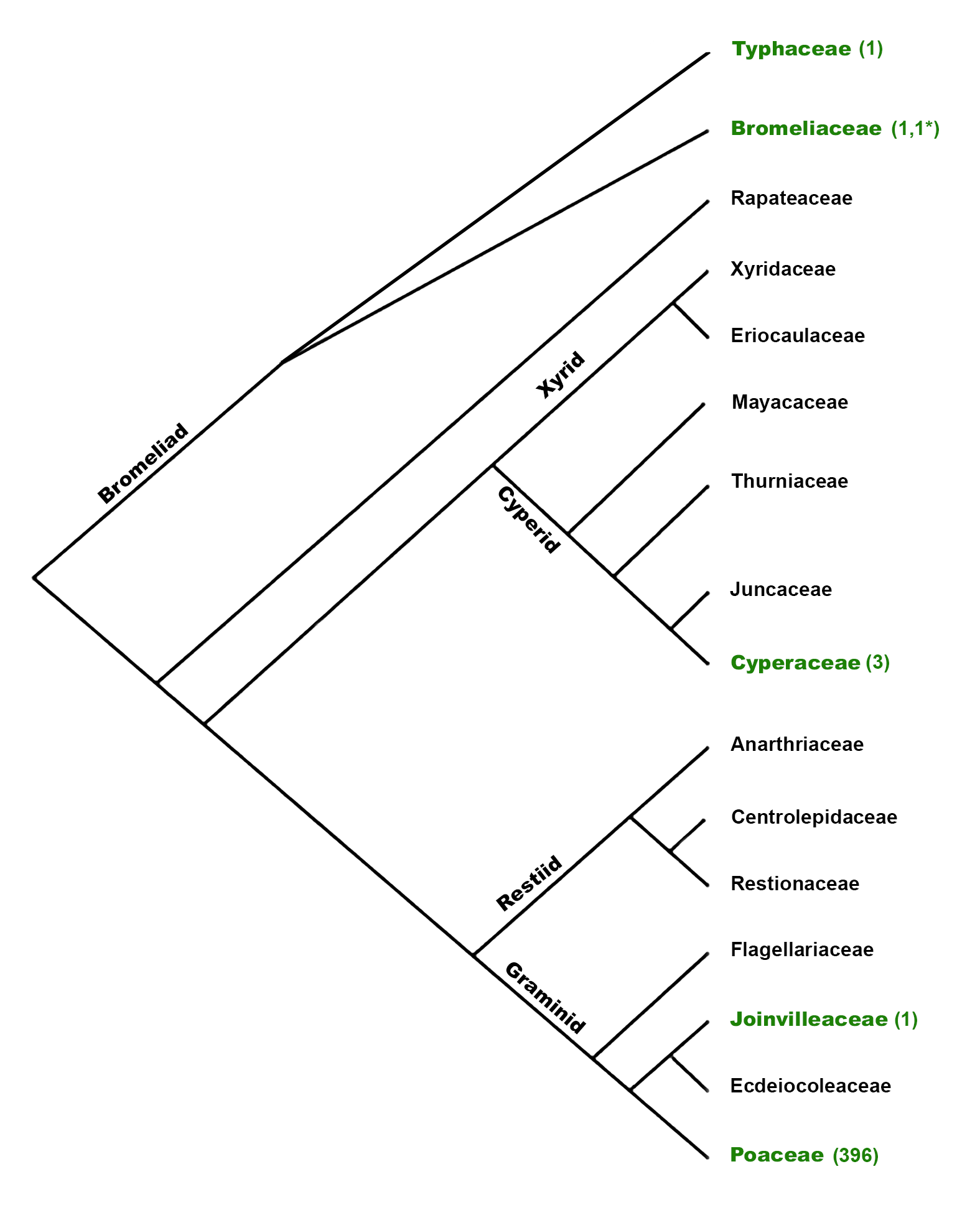

Supplement: S1 Fig — Family names in green indicate the availability of plastome genomes. Numbers indicate available plastome genomes. Asterisks indicate the presence of available but unpublished genome. (TIF) [file pone.0221423.s001.tif]

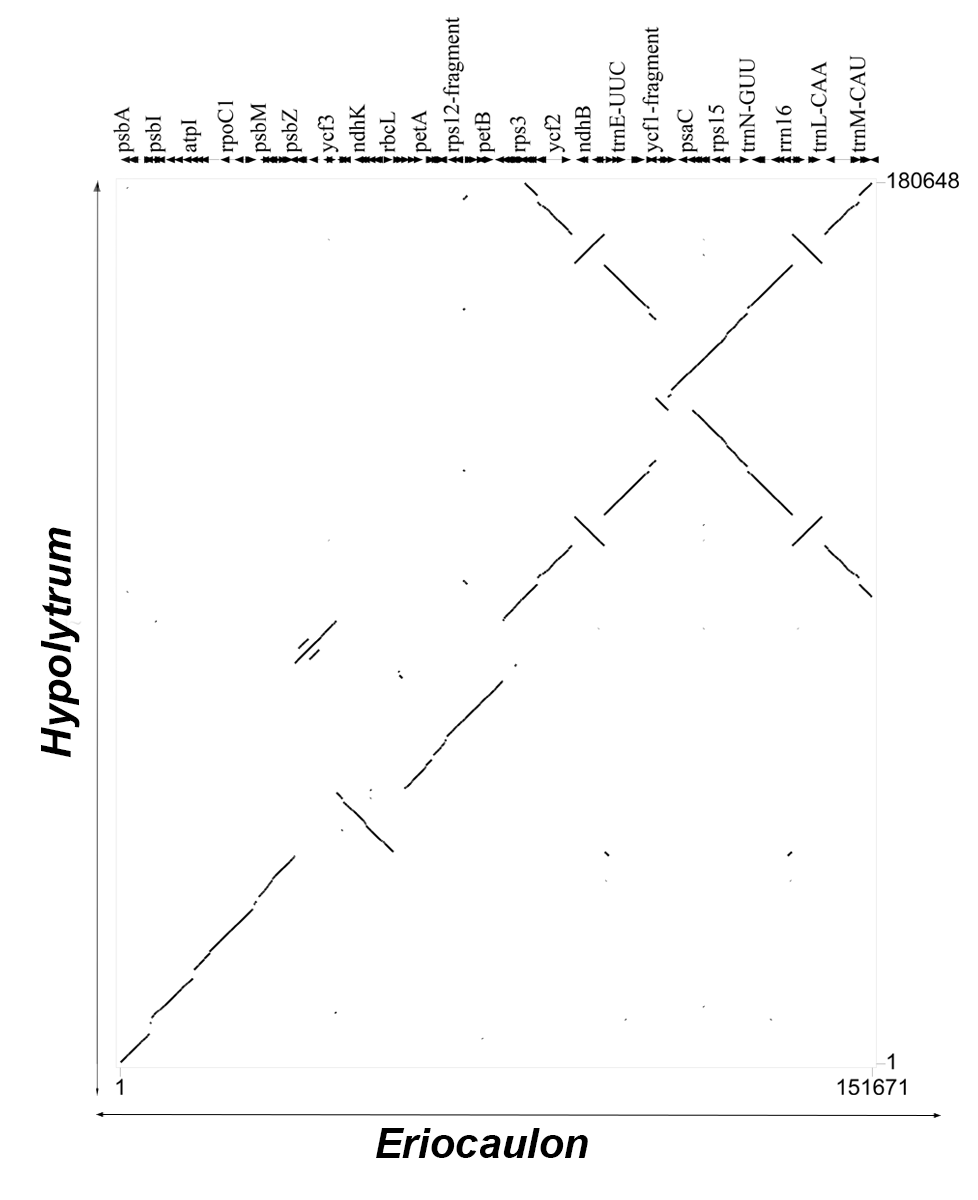

Supplement: S2 Fig — Eriocaulon decemflorum compared to Hypolytrum nemorum. Numbers along the X-axis indicate the coordinates for Eriocaulon and along the Y-axis for Hypolytrum. (TIF) [file pone.0221423.s002.tif]
